# Phytochemistry and Preliminary Assessment of the Antibacterial Activity of Chloroform Extract of *Amburana cearensis* (Allemão) A.C. Sm. against *Klebsiella pneumoniae* Carbapenemase-Producing Strains

**DOI:** 10.1155/2014/786586

**Published:** 2014-03-17

**Authors:** Mirivaldo Barros Sá, Maria Taciana Ralph, Danielle Cristina Oliveira Nascimento, Clécio Souza Ramos, Isvânia Maria Serafin Barbosa, Fabrício Bezerra Sá, J. V. Lima-Filho

**Affiliations:** ^1^Departamento de Biologia, Universidade Federal Rural de Pernambuco, R. Dom Manoel de Medeiros s/n, Campus Dois Irmãos, 52171-900 Recife, PE, Brazil; ^2^Departamento de Ciências Moleculares, Universidade Federal Rural de Pernambuco, R. Dom Manoel de Medeiros s/n, Campus Dois Irmãos, 52171-900 Recife, PE, Brazil; ^3^Departamento de Biofísica, Universidade Federal Rural de Pernambuco, R. Dom Manoel de Medeiros s/n, Campus Dois Irmãos, 52171-900 Recife, PE, Brazil; ^4^Departamento de Morfologia e Fisiologia Animal, Universidade Federal Rural de Pernambuco, R. Dom Manoel de Medeiros s/n, Campus Dois Irmãos, 52171-900 Recife, PE, Brazil; ^5^Laboratório de Microbiologia e Imunologia, Departamento de Biologia, Universidade Federal Rural de Pernambuco, R. Dom Manoel de Medeiros s/n, Campus Dois Irmãos, 52171-900 Recife, PE, Brazil

## Abstract

The chloroform extract of the stem bark of* Amburana cearensis *was chemically characterized and tested for antibacterial activity.The extract was analyzed by gas chromatography and mass spectrometry. The main compounds identified were 4-methoxy-3-methylphenol (76.7%), triciclene (3.9%), **α**-pinene (1.0%), **β**-pinene (2.2%), and 4-hydroxybenzoic acid (3.1%). Preliminary antibacterial tests were carried out against species of distinct morphophysiological characteristics:* Escherichia coli*,* Salmonella enterica *Serotype Typhimurium,* Pseudomonas aeruginosa*,* Staphylococcus aureus*,* Listeria monocytogenes*, and* Bacillus cereus*. The minimum inhibitory concentration (MIC) was determinate in 96-well microplates for the chloroform extract and an analogue of themain compound identified, which was purchased commercially.We have shown that plant's extract was only inhibitory (but not bactericidal) at the maximum concentration of 6900 **μ**g/mL against* Pseudomonas aeruginosa *and* Bacillus cereus*. Conversely, the analogue 2-methoxy-4-methylphenol produced MICs ranging from215 to 431 **μ**g/mL against all bacterial species.New antibacterial assays conducted with such chemical compound against* Klebsiella pneumoniae *carbapenemase-producing strains have shown similarMICresults and minimumbactericidal concentration (MBC) of 431 **μ**g/mL.We conclude that* A. cearensis *is a good source of methoxy-methylphenol compounds,which could be screened for antibacterial activity againstmultiresistant bacteria fromdifferent species

## 1. Introduction

*Amburana cearensis* (Allemão) A.C. Sm. (Fabaceae) is a native plant from Brazilian semiarid region widely used in folk medicine to treat nervous disorders, headaches, asthma, sinusitis, bronchitis, flu, and rheumatic pain, with the stem bark and seeds commonly being consumed as tea or infusion preparations [1–3]. The stem bark is rich in coumarin (1,2-benzopyrone), whose pharmacological properties include anti-inflammatory, antinociceptive, and bronchodilator effects [4–7]. Indeed, four new compounds (p-hydroxybenzoic acid, aiapin, and two stereoisomers of o-coumaric acid glycoside) were recently identified, showing plants' repertory of potential bioactive substances [8]. However, although* A. cearensis *has been broadly used in treatment of respiratory infections, only a few studies report antimicrobial activity in plants' extracts [9, 10], and, therefore, its potential as a source of new antimicrobials was not fully investigated.

The spread of multidrug resistance among bacteria from various sources [11–16] has made natural product researchers increase their effort on screening plant extracts for compounds with broad spectrum of antimicrobial activity. For example,* Tragia involucrata *L.,* Citrus acida* (Roxb. Hook.f.), and* Aegle marmelos *(L.) Correa ex Roxb. showed wide inhibitory action against several multidrug-resistant human pathogens, particularly,* Burkholderia pseudomallei* and* Staphylococcus aureus, *which was related to the high contents of phenolic or polyphenolic compounds in methanol extracts [17, 18]. Likewise, recent antibacterial assays with stem bark ethanol extracts of* A. cearensis* have shown growth inhibition of a broad range of pathogens of veterinary interest [10]. In particular, hospital-acquired infections caused by* Klebsiella pneumoniae* (Enterobacteriaceae) have increased in recent years due to emergence of carbapenemase- producing strains [19, 20]. These bacteria are capable of hydrolyzing carbapenems, penicillins, cephalosporins, and aztreonam [21], and, therefore, the search for new therapeutics is forthcoming.

Here, a preliminary assessment of the antibacterial activity of* A. cearensis* chloroform extracts against human clinical isolates of* K. pneumoniae* carbapenemase-producing strains was conducted. Moreover, the plants' extract was chemically characterized and an analogue of the major compound identified was also tested against bacteria. Although the chemical composition of plant extracts varies under influence of seasonal and climatic conditions [22], in the present study, we report* A. cearensis* as a new source of methoxy-methylphenol compounds with antibacterial activity. These are phenol derivatives with potential applications as antiseptics and biocides. These data are discussed in light of previous studies.

## 2. Material and Methods

### 2.1. Collection and Botanical Identification

The stem bark of* A. cearensis* was collected in the municipality of Salgueiro, Pernambuco, Brazil (latitude 08°04′27′′ south and longitude 39°07′09′′ west). The plant's identification was made by comparing the aerial parts with exsiccate samples (voucher number 46090) deposited in the collection of Vasconcelos Sobrinho Herbarium, Department of Biology, Federal Rural University of Pernambuco, under the care of Dr. Suzene Izidio da Silva.

### 2.2. Plant Extract

Fresh plant material was collected and dried in oven at a temperature range of 45°C to 50°C for 48 h. The dried material was ground in a blade mill to obtain a fine homogeneous powder. This material was weighed and extracted by maceration using chloroform (P.A.) as solvent extractor in the ratio of 1 : 3 (w : v). The resulting mixture remained for 48 hours under agitation every two hours. The extract was filtered and concentrated in a rotary evaporator under reduced pressure at a temperature of 45°C for complete solvent removal. Stock solutions were prepared with extracts using dimethyl sulfoxide (DMSO) as solvent (100 mg/mL), which were kept in a refrigerator at −20°C until use [23].

### 2.3. Analysis by Gas Chromatography Coupled to Mass Spectrometry (GS/MS)

The plant extract was analyzed by GS/MS using a Varian 431-GC chromatograph coupled to a Varian 220-MS mass spectrometer, equipped with a J & W Scientific DB5 fused silica capillary column (30 m × 0.25 mm × 0.25 mm). The temperature of the injector and detector was set at 260°C with the furnace temperature programmed in a range of 60–240°C at 3°C/min. The mass spectra were obtained with a 70 eV electron impact, 0.84 scan/sec* m/z* 40–550. The carrier gas used was helium at a flow rate of 1 mL/min. A stock solution of 2 mg/mL was prepared and 1.0 *μ*L was injected for analyses. The identification of the constituents was carried out by comparison with previously reported values of retention indices, obtained by coinjection of oil samples and C_11_–C_24_ linear hydrocarbons and calculated using the van Den Dool and Kratz equation [24], by direct comparison of the spectra with spectra stored in libraries of equipment (NIST21 and NIST107) as well as with the spectra and retention times of authentic compounds reported previously in the literature for comparison. Subsequently, the MS acquired for each component was matched with those stored in the NIST21, NIST107 mass spectral library of the GC-MS system and with other published mass spectral data [25].

### 2.4. Microorganism and Growth Conditions

Preliminary assessment of antibacterial activity was conducted with bacteria of distinct morphophysiological characteristics, such as* Escherichia coli *(facultative anaerobe, gram-negative, nonencapsulated, extracellular bacteria, Enterobacteriaceae),* Salmonella enterica* Serotype Typhimurium (facultative anaerobe, gram-negative, nonencapsulated, intracellular bacteria, Enterobacteriaceae),* Pseudomonas aeruginosa *(aerobe, gram-negative, extracellular bacteria, non-Enterobacteriaceae),* Staphylococcus aureus *(facultative anaerobe, gram-positive, non-spore-forming, extracellular bacteria),* Listeria monocytogenes *(facultative anaerobe, gram-positive, non-spore-forming, intracellular bacteria),and* Bacillus cereus *(aerobe, gram-positive, spore-forming, extracellular bacteria) belonging to the collection of the Laboratory of Microbiology and Immunology of Federal Rural University of Pernambuco (UFRPE).* Klebsiella pneumoniae* carbapenem-resistant strains (KPC) were obtained from human clinical cases and kindly provided by Dr. Marcia Moraes (University of Pernambuco). Single colonies from fresh cultures were streaked in tubes containing Brain Heart Infusion Agar after growth (37°C/18–24 h) and kept at 8°C until use.

### 2.5. Determination of the Minimum Inhibitory Concentration (MIC) and Minimum Bactericidal Concentration (MBC)

The MIC of the plant extract was determined by the microdilution method [34] in 96-well plates at concentrations ranging from 6.7 to 6900 µg/mL. In addition, the analogue 2-methoxy-4-methylphenol of the main compound identified in plant extracts (4-methoxy-3-methylphenol) was purchased commercially (Sigma) and also investigated. The density of the bacterial suspension was adjusted to approximately 10^5^ CFU/mL, and the results were read after an incubation period of 24 hours at 37°C. The MIC was the lowest concentration causing inhibition of visible growth. In this case, aliquots of 0.1 mL were transferred to plates containing Mueller-Hinton agar. The minimum bactericidal concentration (MBC) was considered the lowest concentration that resulted in no growth after incubation for 24 h at 37°C. All assays were performed in duplicate.

## 3. Results and Discussion

The Brazilian floras are worldwide known as a source of biologically active compounds with biodegradable properties, and, therefore, screening of plant extracts is often a first step procedure for isolation of phytochemicals [23]. However, studies that correlate antibacterial activity of extracts to the presence of specific compounds are rare. For instance, ethanol extracts of the stem bark of* A. cearensis* were reported to be inhibitory against* Staphylococcus epidermidis, S. aureus, Klebsiella* spp.,* Salmonella* spp.,* Enterobacter aerogenes, Streptococcus pyogenes, Proteus mirabilis, Pseudomonas aeruginosa,* and* Shigella flexneri, *but no chemical characterization was performed [10]. Here, we report the phytochemistry and preliminary assessment of* A. cearensis *chloroform extracts as a source of novel antimicrobials against multiresistant* Klebsiella* strains.

**Figure 1:**
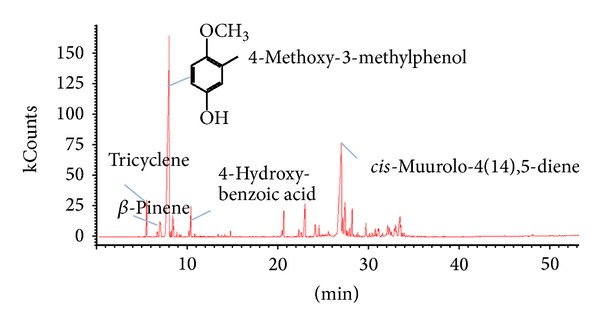
Chromatogram of chloroform extract of the stem bark of* A. cearensis*.

The GC/MS data showed a major peak at the retention time of 7.8 min with relative concentration of 76.7% (Figure 1). The interpretation of the mass spectra in comparison with spectra reported in the literature led to identification of the benzenoid 4-methoxy-3-methylphenol. Other compounds included tricyclene (3.9%), *α*-pinene (1.0%), *β*-pinene (2.2%), and 4-hydroxybenzoic acid (3.1%). The complete list of compounds identified in plants' extract is reported in Table 1. Several compounds have been identified in the bark of* A. cearensis*, such as trans -3,4-methyl dimetoxicinamato,* cis*-3,4-dimethoxy-methyl cinnamate, 3-methoxy-4-hydroxy-methyl cinnamate, 4-hydroxy-methyl benzoate, 3,4-dihydroxy methyl benzoate, 3-hydroxy-4-methoxy methyl benzoate, catechol, guaiacol, a-ethoxy-p-cresol, benzenemethanol 4-hydroxy, 4-methoxy-methylphenol, 2,3-dihydrobenzofuran, and anthraquinone (chrysophanol), with predominance of coumarins being often reported [35].

The initial screening for antibacterial activity has shown that plants' chloroform extract was only inhibitory against* P. aeruginosa* and* B. cereus* at the highest concentration of 6900 µg/mL (Table 2).* P. aeruginosa* is responsible for different etiological processes in immunocompetent and immunocompromised patients in hospitals, whereas* B. cereus* is an opportunistic pathogen [36, 37]. On the other hand, the analogue compound 2-methoxy-4-methylphenol showed broad spectrum activity against all bacteria tested (Table 3). Indeed, the MIC ranged from 215 to 431 µg/mL against* K. pneumoniae* carbapenemase-producing strains being also bactericidal at 431 µg/mL (Table 3). The carbapenems imipenem, meropenem, and ertapenem are often used as last therapeutic choices against gram-negative multidrug-resistant bacteria [38]. Moreover, these bacteria usually show resistance to aminoglycosides and fluoroquinolones due to the presence of gene* gnr* and *bla*_kpc_ [39]. Thus, the risk of nosocomial infections is higher for patients in hospital's intensive care units [40, 41].

According to PubChem Compound Database, 4-methoxy-3-methylphenol is also known as 14786-82-4, AG-D-93185, NSC168522, AC1L6RKC, SureCN263095, and 4-methoxy-3-methylphenol [42]. Although anticancer assays were previously carried out with the compound, it was reported to be inactive against tumor model L1210 leukemia in mice [42]. While we were not aware of previous antimicrobial studies conducted with 4-methoxy-3-methylphenol, we hypothesize that the action mechanism of the molecule against bacteria is possibly due to 3-methylphenol compound (*m*-cresol) well known as an oxidizing agent [43]. Yet,* m*-cresol is a methyl derivative of phenol that has been used as precursor of amylmetacresol present in commercial antiseptic formulation.

The array of chemical compounds in* A. cearensis* extracts varies despite the type of solvent used (Table 4). Accordingly, we have shown that chloroform extracts of* A. cearensis* are good sources of methoxy-methylphenol compounds with antibacterial activity against several bacterial species and clinical isolates of multidrug-resistant* K. pneumonia. *Thus, the plants' chloroform extract could be exploited as a source of antiseptics or biocides against drug-resistant bacteria from different species.

## Figures and Tables

**Table 1:** Chemical composition of the chloroform extract of the stem bark of *Amburana cearensis. *

Compounds^a^	Relative area (%)	RI^b^	RI^c^
Tricyclene	3.9	919	921
**α**-Pinene	1.0	931	932
**β**-Pinene	2.2	974	974
6,10-Dodecatrien-1-ol, 3,7,11-trimethyl	2.3^d^	—	—
4-Methoxy-3-methylphenol	76.7^d^	—	—
Terpinolene	0.3	1087	1088
1,3,8-*p*-Menthatriene	0.5	1107	1108
4-Hydroxybenzoic acid	3.1^d^	—	—
Longifolene	1.7	1405	1407
*cis*-Muurolo-4(14),5-diene	3.2	1462	1465

Total	94.9	—	—

^a^Compounds are listed in order of their elution from a DB-5 column; ^b^RI = retention indices relative to C_7_–C_30_ *n*-alkanes; ^c^RI = retention indices from the literature.^ d^Identified by direct comparison of the spectra with spectra stored in libraries of equipment as well as with the spectra and retention times of authentic compounds reported previously in the literature for comparison.

**Table 2:** Antibacterial activity of the chloroformextract of *A. cearensis* and the analogue 2-methoxy-4-methylphenol.

Bacterial species	Chloroform extract		2-Methoxy-4-methylphenol		Ciprofloxacin	
	MIC*	MBC	MIC	MBC	MIC	MBC
	µg/ mL					
*Salmonella enterica *Typhimurium	—	—	215	431	<6.7	<6.7
*Escherichia coli *	—	—	215	431	<6.7	<6.7
*Pseudomonas aeruginosa *	>6900	—	431	>6900	<6.7	<6.7
*Bacillus cereus *	>6900	—	431	3450	<6.7	107
*Listeria monocytogenes *	—	—	215	862	<6.7	<6.7
*Staphylococcus aureus *	—	—	215	862	<6.7	<6.7

*MIC: minimum inhibitory concentration; MBC: minimum bactericidal concentration.

**Table 3:** Antibacterial activity of 2-methoxy-4-methylphenol against *Klebsiella pneumoniae* carbapenemase-producing strains.

*K. pneumonia* strains	2-Methoxy-4-methylphenol		Ciprofloxacin	
	MIC*	MBC	MIC	MBC
	(µg/mL)			
KPC 201	215	431	<6.7	<6.7
KPC 199	215	431	<6.7	<6.7
KPC +	215	431	<6.7	215
KPC 278	215	431	<6.7	431

*MIC: minimum inhibitory concentration; MBC: minimum bactericidal concentration.

**Table 4:** Phytochemicals reported in extracts of *A. cearensis. *

Part used	Solvent	Secondary metabolites	Antibacterial activity	Reference
Stem bark	Ethanol	Not reported	*Staphylococcus epidermidis *	[9]
Stem bark	Ethanol	Not reported	Cocci strains, Enterobacteria, non-fermenting bacteria	[10]
Stem bark and leaves	Ethanol	Anthocyanins, anthocyanidins, flavones, chalcones, aurones, leucoanthocyanidins	Not reported	[11, 13]
Seeds	Butanol and hydroethanol	Flavonoids, proanthocyanidins, anthocyanins, carotenoids	Not reported	[26]
Aerial parts and xylopodium	Ethanol	Protocatechuic acid, vanillic acid, coumarin, amburoside	Not reported	[27]
Resin	Methanol and chloroform	Chalcone, 2′,4,4′-trihydroxy chalcone (isoliquiritigenin a) (1), 2′,4′, dihydroxy-3′,4′-methoxychalcone (2), 7,8,3′,4′-tetramethoxy isoflavone, 2′,4,4′-trihydroxy (isoliquiritigenin) (1); 2′,4′,dihydroxy-3′,4′-methoxy (2),7,8,3′,4′-tetramethoxy isoflavone	Not reported	[28]
Stem bark	Ethanol	Coumarin and phenolic compounds (isokaempferide and amburoside)	Not reported	[29]
Wood powder	Hydroethanol	1-Dodecanol; 2-ethyl-hexane acid; dihydrocoumarin; coumarin (1,2- benzopyrone)	Not reported	[30]
Stem bark	Ethanol	Isoflavonoid (afromorsin)	Not reported	[31]
Stem bark	Hexane and chloroform	Cumarina Coumarin (1,2 - benzopirona) (1,2-benzopyrone)	Not reported	[32]
Trunk bark	Ethanol	Coumarin and vanillic acid	Not reported	[33]
